# The geographical distribution and socioeconomic risk factors of COVID-19, tuberculosis and leprosy in Fortaleza, Brazil

**DOI:** 10.1186/s12879-023-08627-9

**Published:** 2023-10-06

**Authors:** A. T. Taal, J. G. Barreto, G. dos Santos de Sousa, A. Medeiros da Rocha, N. N. Lima Ferreira, J. A. Menezes da Silva, D. C. Hinders, W. H. van Brakel, J. H. Richardus, D. J. Blok

**Affiliations:** 1 NLR, Amsterdam, The Netherlands; 2 Erasmus MC, University Medical Center Rotterdam, Rotterdam, The Netherlands; 3 Federal University of Pará, Belém, Brazil; 4 Fortaleza Municipal Health Secretariat, Fortaleza, Brazil; 5 NHR Brasil, Fortaleza, Brazil

**Keywords:** COVID-19, Tuberculosis, Leprosy, Geographical distribution, Socioeconomic risk factors

## Abstract

**Background:**

Fortaleza (Brazil) is high endemic for coronavirus disease 2019 (COVID-19), tuberculosis (TB) and leprosy. These three diseases share respiratory droplets through coughing or sneezing as the main mode of transmission but differ in incubation time, with COVID-19 having a short and leprosy a long incubation time. Consequently, contacts of a patient are at higher risk of infection and developing these diseases. There might be scope for combined preventive measures, but a better understanding of the geographical distribution and relevant socioeconomic risk factors of the three diseases is needed first. This study aims to describe the geographic distribution of COVID-19, TB and leprosy incidence and to identify common socioeconomic risk factors.

**Methods:**

The total number of new cases of COVID-19, TB and leprosy, as well as socioeconomic and demographic variables, were retrieved from official registers. The geographical distribution of COVID-19, TB and leprosy rates per neighbourhood was visualised in Quantum GIS, and spatial autocorrelation was measured with Moran’s I in GeoDa. A spatial regression model was applied to understand the association between COVID-19, TB, leprosy rates, and socioeconomic factors.

**Results:**

COVID-19 and TB showed a more homogenous distribution, whereas leprosy is located more in the south and west of Fortaleza. One neighbourhood (Pedras) in the southeast was identified as high endemic for all three diseases. Literacy was a socioeconomic risk factor for all three diseases: a high literacy rate increases the risk of COVID-19, and a low literacy rate (i.e., illiteracy) increases the risk of TB and leprosy. In addition, high income was associated with COVID-19, while low income with TB.

**Conclusions:**

Despite the similar mode of transmission, COVID-19, TB and leprosy show a different distribution of cases in Fortaleza. In addition, associated risk factors are related to wealth in COVID-19 and to poverty in TB and leprosy. These findings may support policymakers in developing (partially combined) primary and secondary prevention considering the efficient use of resources.

**Supplementary Information:**

The online version contains supplementary material available at 10.1186/s12879-023-08627-9.

## Introduction

For years, Brazil has had a high burden of infectious diseases [1]. As of August 2023, it is the world’s sixth leading country in the number of coronavirus disease 2019 (COVID-19) cases, with 37,717,062 confirmed cases and 704,659 reported deaths up to 16 August 2023 [2]. In addition, Brazil is also listed as the second highest burden country for leprosy and the 20th for tuberculosis (TB), with 14,962 new cases of leprosy and 78,057new cases of TB reported to the Ministry of Health in 2022 [3, 4].

These three diseases share a human-to-human primary transmission mode, via respiratory droplets emitted by coughing or sneezing, though have different incubation times. COVID-19 is caused by the severe acute respiratory syndrome coronavirus 2 (SARS-CoV-2) and is a systemic disease primarily affecting the lungs. The incubation time is two to 14 days, and most people infected with the virus will experience mild to moderate symptoms, including coughing, fever and malaise. In severe cases, however, people can have difficulty breathing and neurologic complications, eventually leading to death [5]. TB is caused by *Mycobacterium tuberculosis*, which affects the lungs as well. It can take two to 10 weeks before infection symptoms appear. However, most infected people never develop active disease and remain with latent infection. In active TB, common symptoms are a persistent productive cough for more than three weeks along with fever, fatigue, and unintentional weight loss; and if left untreated, TB can lead to death [6]. Conversely, leprosy is caused by *Mycobacterium leprae* and affects the skin and peripheral nerves. It has a long incubation time varying from 2 to 20 years. Known symptoms of leprosy are skin lesions with decreased or altered sensitivity and enlarged nerves. Delay in diagnosis and lack of treatment can lead to lifelong physical disabilities and social discrimination [7]. For all three diseases, contacts of an infectious person, especially household contacts, are at higher risk of infection and developing disease [5, 8, 9].

Fortaleza, the capital city of Ceará state in northeastern Brazil, is highly endemic for COVID-19, TB and leprosy. It reported the first COVID-19-positive case in March 2020, and since then 716,274 confirmed cases and 17,485 deaths due to COVID-19 have been reported up to March 2023 [10]. For TB, 1,476 new positive cases were reported in 2019, 1,173 in 2020, 1,958 in 2021 and 2,232 in 2022; for leprosy, 545 new cases were reported in 2019, 465 in 2020, 614 in 2021 and 551 in 2022 [11]. The decrease in the number of new cases of TB and leprosy in 2020 is likely an operational effect of COVID-19. Beginning in April 2020, the Ceará health department recommended that everyone, especially those with known risk factors, seek basic care at the first COVID-19 sign or symptom. This generated a great demand for primary health centres, which contributed to the health system’s collapse [12]. Together with social isolation regulations, a lockdown from May 2020 to 2021, and the public fear of becoming infected with COVID-19 in health facilities, this resulted in COVID-related under-reporting of new cases of TB and leprosy in Fortaleza [13, 14].

The geographical distribution of these diseases within an area is likely associated with socioeconomic risk factors. For example, at the beginning of the COVID-19 pandemic, the distribution of cases was restricted to neighbourhoods in the central of Fortaleza, and factors associated with higher COVID-19 incidence rates could be related to better socioeconomic conditions, access to testing and care and population mobility. Studies indicated that the higher the social inequality (measured by the Gini index), the higher the COVID-19 notifications at the municipal level [15–17]. In addition, higher incidences were seen in populations with a high Human Development Index (HDI), higher income and a higher nurse ratio per 1,000 population. After self-isolation, lockdown and increased physical distance measures were put in place, risk factors shifted relative to the vulnerability of the population, including large household size, low literacy, unemployment, poverty, less access to running water and sanitation, living in an urban area, and high population density [15, 17, 18]. Known socioeconomic risk factors for TB and leprosy are also commonly related to poverty and social vulnerability [19–21].

Preventive measures are required to interrupt transmission and reduce the burden of these three airborne diseases in Fortaleza. For COVID-19 and TB, primary prevention consists of good ventilation, good hygiene, facemasks that can prevent the further spread of aerosols, and vaccination to strengthen the host’s immune system. For leprosy, however, no primary prevention methods are in place due to long incubation time and not knowing when someone is infectious. Therefore, secondary prevention is important to reduce the impact of diseases and focuses on early detection of new cases. For COVID-19, this is testing and self-isolation for contacts of positive COVID-19 cases [4, 13]. The current strategy for early case finding and prevention of leprosy includes active case finding, screening of contacts of leprosy patients for signs and symptoms of leprosy and, if eligible (no signs or symptoms), administration of a single dose of rifampicin of post-exposure prophylaxis (SDR-PEP) [22]. For TB, the END TB strategy, adopted by the World Health Organization (WHO), is focused on preventing TB infection and stopping progression from infection to disease in at-risk groups such as HIV-positive and household contacts [23]. This is done via community-based active case finding, screening for TB signs and symptoms, testing for latent TB infection and preventive treatment. In Brazil, preventive treatment for latent TB infections consists of four months of daily rifampicin (4R), or six to nine months of daily isoniazid (6/9H). For children below 10 years and adults above 50 years old, 4R is recommended [24].

Despite the similarities in primary and secondary prevention of the three diseases, Brazil currently has separate COVID-19, TB and leprosy disease control programmes. Fortaleza reported a shortage of qualified primary care professionals as the 115 primary healthcare centres (PHCs) covered only 63% of the population in 2020 [25–27]. Allocating available (human) resources efficiently is crucial. As the diseases share certain characteristics, combined prevention approaches might be considered. However, further understanding of the geographical distribution at a smaller scale and underlying socioeconomic factors is required. Hence, this study aims to describe the geographic distribution of COVID-19, TB and leprosy incidence and to identify common associated socioeconomic risk factors. The findings of this study could provide insights for potentially targeted and integrated primary and secondary prevention.

## Methods

### Study area

Fortaleza is the capital city of Ceará state, located in north-eastern Brazil. It has an area of 312 km^2^, with an estimated population of 2.4 million in 2022, resulting in a highly densely populated city of 7,775 inhabitants/km^2^. Fortaleza ranks 15th among the Brazilian cities with the highest income inequality, with a Gini index score of 0.6 and a municipal HDI of 0.754 in 2010 [28]. The proportion of urban population living in slums (informal housing) was 16% in 2019. The average number of hospital beds and doctors per 100,000 population was 377 and 295, respectively, for the same year [29].

### Data sources

This study collected relevant data from various sources, including official websites, reports and the preventive trial coordinated by NHR Brasil (PEP + + project). The total number of Polymerase Chain Reaction-positive cases of COVID-19 and the total number of deaths due to COVID-19 registered from 17 to 2020 to 4 September 2022 per neighbourhood were retrieved from the weekly COVID-19 report disseminated by the municipal health office in Fortaleza on 13 September 2022 [10]. Two years of COVID-19 data was collected to correct for the effect of the unequal access to testing in the first months of the pandemic. For TB and leprosy, we retrieved seven years of (available) incidence data from the National Notifiable Diseases Information System (SINAN). For TB, this was the total number of confirmed TB cases registered from 2015 to 2021 per neighbourhood [29]. For leprosy, this was the total number of new leprosy cases registered from 1 2014–2020 [31]. A participatory mapping approach was used to verify the locations of the leprosy patients because incorrect or incomplete addresses are often registered in SINAN. Participatory mapping included asking health workers of the PHCs in Fortaleza to pinpoint the residence of leprosy patients on a digital map and collect the corresponding geocoordinates with the mobile application MapIt (version 7.6.0, https://mapitgis.com/). The data points were uploaded in Quantum Geographic Information System (QGIS) version 3.22.10 (QGIS Developer team, Bialowieza [2022]) and aggregated per neighbourhood. Variables related to demographic and socioeconomic were retrieved from the 2010 census, as this is the most recent publicly available census data [32]. We selected the population, household size, number of households with their own bathroom and toilet, mean monthly income of people > 10 years old, age, gender, and literate people above five years old per census tract. The spatial data was organised in shapefiles by neighbourhood as retrieved from the Brazilian Institute of Geography and Statistics (IBGE; https://www.ibge.gov.br) [33].

### Spatial analysis

#### Distribution

The geographical distribution of COVID-19, TB and leprosy cases per neighbourhood was visualised in QGIS using the ‘points in polygons’ tool. For each disease, the incidence rate per neighbourhood was calculated as the total number of cases divided by the population in the neighbourhood and multiplied by 100,000. We created choropleth maps of the incidence rates and of the distribution of population, population density, mean household size, mean monthly household income, proportion of literate people above five years (i.e., literacy rate), and proportion of households with their own bathroom and toilet per neighbourhood.

#### Spatial autocorrelation

The Moran’s I statistic with Empirical Bayes (EB) standardisation was used to estimate the spatial autocorrelation of COVID-19, TB and leprosy incidence rates and of different variables using the software GeoDa 1.18. Empirical Bayes standardisation was applied to correct the variance in population densities [34]. The incidence rate was used as the ‘event variable’ and the total population as the ‘base event’, with queen contiguity and 99,999 permutations to account for randomness.

#### Spatial regression

Spatial regression was applied to understand the association between COVID-19, TB, leprosy rates and socioeconomic factors. For each disease, we first applied ordinary least squares (OLS) using GeoDa 1.18. The results of the OLS will determine whether to accept the OLS model or continue with the spatial lag model (SLM) or spatial error model (SEM) [34].

The OLS estimates the parameters in the regression model by minimising the sum of the squared residuals. We evaluated the model based on multicollinearity among the variables, normality of the errors (Jarque-Bera test), heteroskedasticity, and the Lagrange multiplier (LM) test for OLS residuals to decide whether to incorporate a spatial component. SLM is applied if the robust LM lag is significant, and SEM if the LM error is significant.

For each individual disease, we first applied OLS and included population density, the proportion of households with 3–5 persons and the proportion of households with ≥ 6 persons, the proportion of households with monthly household income < minimum wage, the proportion of households with their own bathroom and toilet, female proportion, proportion of population 15–65 years old and > 65 years old, and literacy rate as independent variables. The output of the OLS models showed multicollinearity (value > 300) for all three diseases, indicating that variables are highly correlated and the regression results are not stable (See Table S2 in Supporting Information). For COVID-19 and leprosy, the Jarque-Bera and the heteroskedasticity tests were strongly significant (p-value < 0.05), suggesting a non-normal distribution of the error term and non-constant error variances. Finally, the Lagrange multiplier test was only significant in the leprosy model and the lag version of LM, indicating spatial dependence [29]. Therefore, we used the SLM model for leprosy and kept the OLS model for COVID-19 and TB. In this study, log-likelihood was used to evaluate the goodness of fit; the higher the value (positive), the better the fit.

## Results

### Geographical distribution and moran’s i for COVID-19, tuberculosis and leprosy

**Fig. 1:**
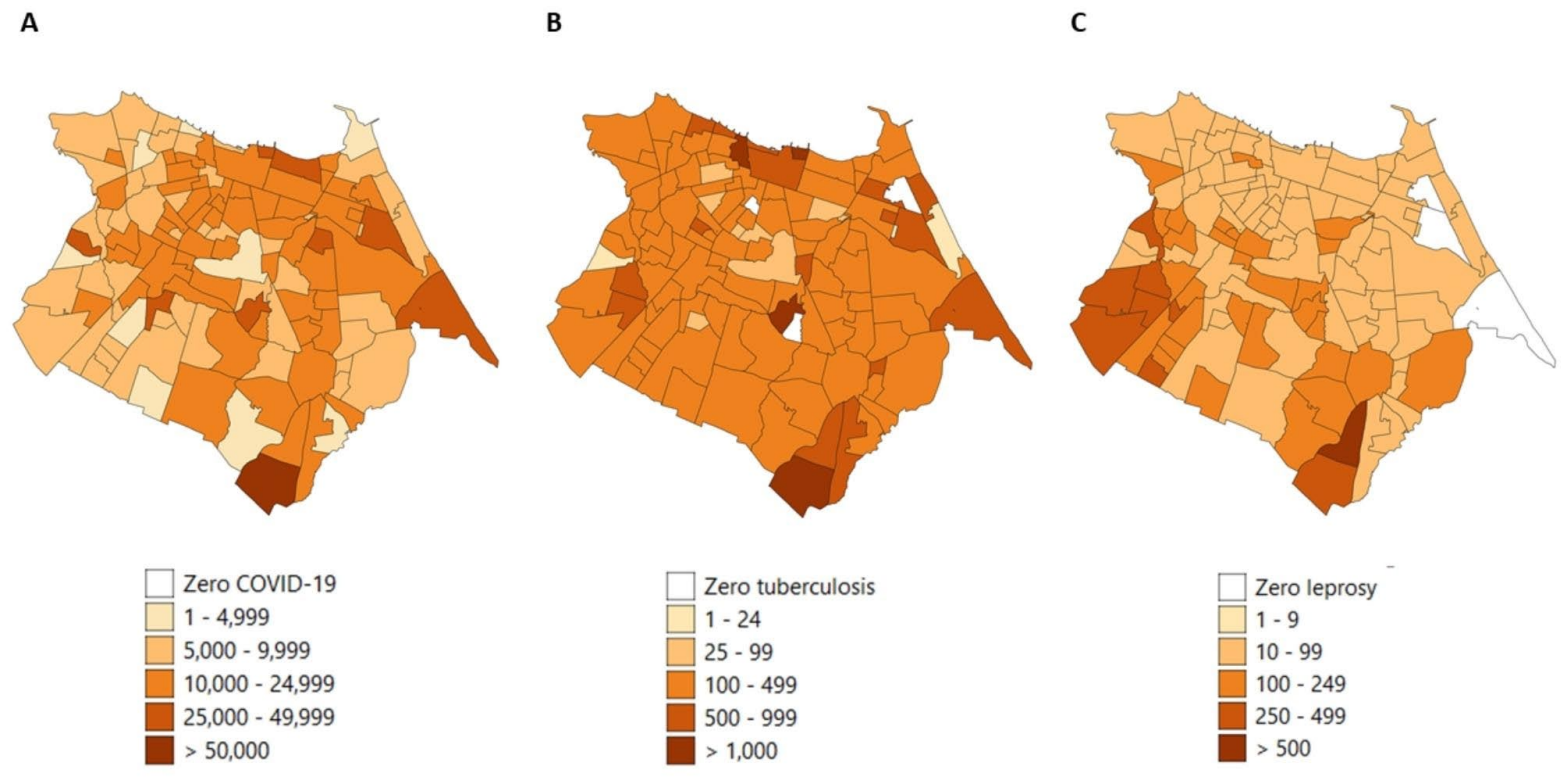
The distribution of raw incidence rates per 100,000 population for COVID-19 **(A)**, tuberculosis **(B)** and leprosy **(C)**

Figure 1 shows the geographical distribution of the raw incidence rates per 100,000 population for the three diseases. We identified one neighbourhood (Pedras) in the southeast that is highly endemic for all three diseases. This neighbourhood has a cumulative incidence rate of 58,621 positive COVID-19 cases per 100,000 population from January 2020 to September 2022 (Fig. 1A), 2,366 positive TB cases per 100,000 population from 2015 to 2021 (Fig. 1B), and 270 leprosy cases per 100,000 population from 2014 to 2020 (Fig. 1C). Two neighbourhoods with high incidences of COVID-19 and TB and zero reported leprosy cases were identified in the east (Fig. 1A and B). Moreover, high incidence rates of leprosy were mainly found in the southwest (Fig. 1C).

Descriptive and Global Moran’s I statistics were calculated for the three diseases (Table 1). COVID-19, TB, and leprosy incidence rates were significantly spatial autocorrelated with a Moran I value of 0.121 (p = 0.01), 0.110 (p = 0.02) and 0.412 (p = 0.001), respectively.

**Fig. 2:**
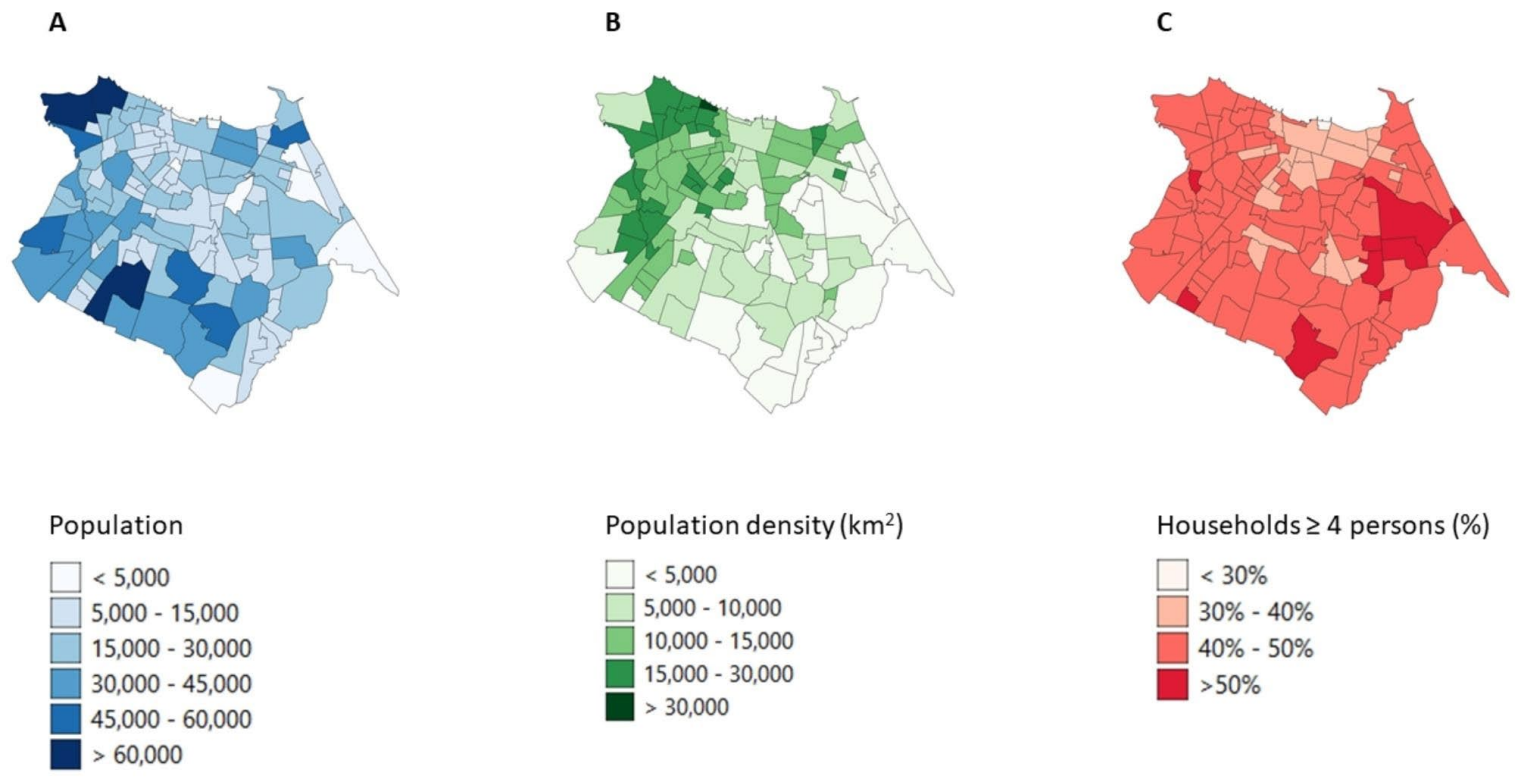
The distribution of population **(A)**, population density per km^2^**(B)**, and mean household size **(C)** per neighbourhood

**Fig. 3:**
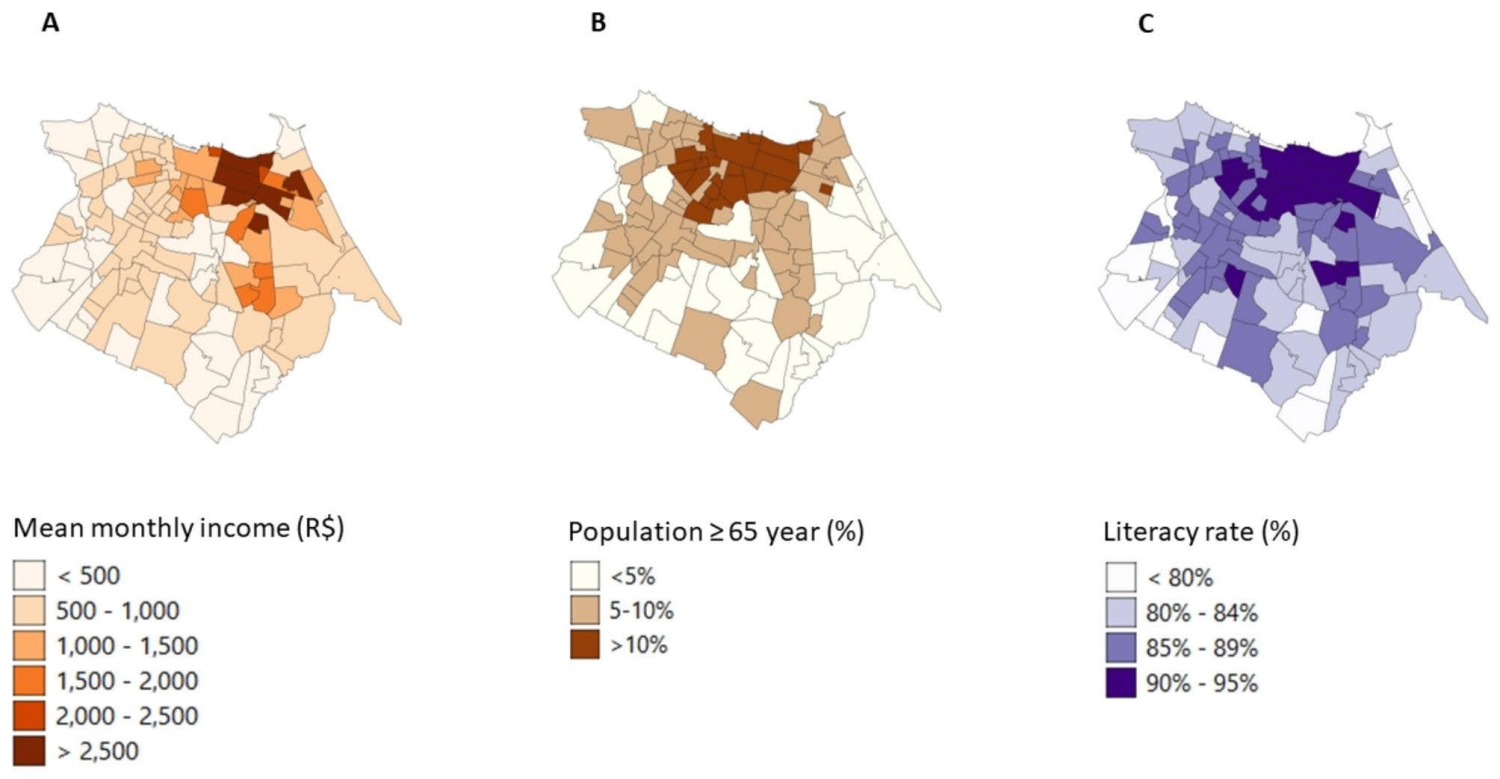
The distribution of monthly income per household in Brazilian Reals **(A)**, proportion of population ≥ 65 years **(B)**, and literacy rate **(C)**

**Table 1:** Descriptive statistics and global Moran’s I for COVID-19, tuberculosis and leprosy incidence rates, and socioeconomic risk factors

	Unit	Median	IQR [Q1-Q3]	SD	Moran’s I
COVID-19 rate	per 100,000 population	10,527	[7759–15,320]	7975.25	0.121*
TB rate	per 100,000 population	337	[201.5-453.5]	298.02	0.110*
Leprosy rate	per 100,000 population	81	[48.5–119]	97.82	0.412***
Population density	per km^2^	10,892	[6041.8-13977.4]	5646.68	0.528***
1–2 persons per household	% of households	0.30	[0.28–0.33]	0.05	0.158**
3–5 persons per household	% of households	0.59	[0.57–0.61]	0.04	0.111*
6 + persons per household	% of households	0.10	[0.09–0.11]	0.02	0.119*
Own bathroom and toilet	% of households	0.95	[0.91–0.97]	0.06	0.117*
Mean monthly household income	Brazilian reais (R$)	619.67	[465.7-970.2]	689.03	0.195**
No wage or < minimum wage	% households	0.64	[0.50–0.77]	0.20	0.099*
> minimum wage^1^	% households	0.35	[0.23–0.50]	0.20	0.293***
Female	% of population	0.53	[0.52–0.54]	0.02	0.126**
< 15 years	% of population	0.21	[0.18–0.25]	0.05	0.113**
≥ 65 years	% of population	0.07	[0.05–0.09]	0.03	0.222***
Literacy rate	% of population	0.87	[0.83–0.90]	0.05	0.125**

^1^ The minimum wage is R$ 510 per month

* p < 0.05, ** p < 0.01, *** p < 0.001

### Geographical distribution and moran’s i for socioeconomic risk factors

Figures 2 and 3 shows the population distribution, population density, mean household size, mean monthly income, proportion of population 65 years old of age and older, and literacy rate. Larger populations and population densities are identified in the west of Fortaleza, compared to the east (Fig. 2A and B). The neighbourhoods in the north, showing high raw incidence rates for COVID-19 (Fig. 1A) and low raw incidence rates for leprosy (Fig. 1C), have a higher mean monthly household income (> R$ 1,500) (Fig. 3A), a larger proportion of population of 65 years and older (Fig. 3B), and a higher literacy rate (Fig. 3C), compared to the neighbourhoods in the south and west. The two southernmost neighbourhoods with high raw incidence rates for all three diseases (Fig. 1A-C) have a small population and population density (Fig. 2A and B), low mean monthly income (Fig. 3A) and low literacy rate (Fig. 3C). Descriptive and Global Moran’s I statistics were also calculated for the various socioeconomic risk factors and were all significant (Table 1).

**Table 2:** Spatial regression analysis of COVID-19, tuberculosis and leprosy incidence rates

	Covid-19 (OLS)		TB (OLS)		Leprosy (SLM)	
R-squared	0.66		0.69		0.48	
Log likelihood	-1173		-776		-678	
Variable	Coefficient	Probability	Coefficient	Probability	Coefficient	Probability
Constant	-100,272	0.03	6810	< 0.001	2028	0.002
*COVID-19 rate*			0.03	< 0.001	0.002	0.16
*TB rate*	21.31	< 0.001			-0.07	0.09
*Leprosy rate*	9.49	0.09	-0.29	0.14		
*Household size 1–2 person(s)*	Ref		Ref		Ref	
*Household size 3–5 persons*	10,379	0.51	-340	0.54	-15	0.94
*Household size ≥ 6 persons*	-66,065	0.04	1416	0.23	-475	0.31
*Monthly income ≥ minimum wage*	Ref		Ref		Ref	
*Monthly income < minimum wage*	-19,511	< 0.001	573	0.003	139	0.07
*Own bathroom & toilet*	68,460	0.07	-3516	0.004	-2094	< 0.001
*Female*	33,842	0.60	-3195	0.02	1482	0.11
*age < 15 years*	Ref		Ref		Ref	
*Age 15–64 years*	-56,063	0.22	-2096	0.21	961	0.14
*Age ≥ 65 years*	-142,259	0.004	7846	< 0.001	803	0.26
*Literacy rate*	93,012	0.007	-4591	< 0.001	-1725	< 0.001
*Lag coefficient* ^*1*^					0.55	< 0.001

^1^ The Lag coefficient reflects the spatial dependence inherent in the sample data, measuring the average influence on observations by their neighbouring observations

### Spatial linear regression

We assessed the relationship between the incidence rates of COVID-19, TB and leprosy and socioeconomic risk factors using an OLS model for COVID-19 and TB and an SLM model for leprosy (Table 2). All three models had a negative log likelihood of more than 500. In the COVID-19 model, a higher TB rate and a higher proportion of literacy in the neighbourhood had a significant positive effect on COVID-19 incidence. In contrast, a lower proportion of household size > 6, low monthly income (< minimum wage) and age ≥ 65 years in the neighbourhood had a significant negative effect. In the TB model, a higher COVID-19 rate, a higher proportion of low monthly income (< minimum wage) and age ≥ 65 years had a significant positive effect on TB incidence, while a lower proportion of owning a bathroom and toilet, and females and low literacy rate had a significant negative effect. In the leprosy model, a lower proportion of people with their own bathroom and toilet and a low literacy rate (i.e., high illiteracy rate) in the neighbourhood had a significant negative effect on leprosy incidence.

## Discussion

This study showed that although COVID-19, TB and leprosy share the same primary transmission mode (human-to-human via respiratory droplets emitted by coughing or sneezing), the geographical distribution of the three diseases and the associated risk factors are different. We found significant clustering of COVID-19, TB and leprosy cases in Fortaleza. COVID-19 and TB cases were distributed throughout Fortaleza and showed a similar distribution, whereas leprosy was found in the south and west. One neighbourhood was identified as highly endemic for all three diseases and had a low mean monthly income and low literacy rate. COVID-19, TB and leprosy shared one common socioeconomic risk factor: literacy rate. The effect of literacy rate on the incidence rate, however, was different. In addition, monthly income (< minimum wage) and ≥ age 65 years were shared as common socioeconomic risk factors by COVID-19 and TB, while not owning one’s own bathroom and toilet was shared by leprosy and TB.

Comparing the geographic distribution of the three diseases, we found high incidence rates of COVID-19 and TB in the northern, central and eastern regions of the city. A similar distribution was found in previous studies in Fortaleza [19, 35, 36]. High incidence rates of leprosy cases were found in neighbourhoods in the south and west, similar to the study of Lima et al. (2015) [37]. The neighbourhoods (Pedras and Ancuri) in the southeast that are high endemic for all three diseases form an industrial area with many factories where people work closely together. Pedras is primarily known for its high social vulnerability.

We found weak clustering of COVID-19 and TB cases and strong clustering of leprosy cases. Although all three diseases are transmitted via aerosols through coughing or sneezing, the level of exposure and time needed before the onset of symptoms are different. In COVID-19, a susceptible person can develop symptoms in 2–17 days after short contact with an infected person, whereas in leprosy, prolonged and frequent contact with an infected person is needed before the first symptoms appear after 2–10 years [38, 39]. Therefore, COVID-19 can spread more efficiently throughout the area, showing a more homogenous distribution (weak clustering) than leprosy, which has a heterogeneous distribution (strong clustering).

In this study, we indicated that literacy rate was associated with disease risk. The association of this risk factor with incidence was positive for COVID-19, meaning that literate people had a higher risk of being notified positive for COVID-19. It was negative for TB and leprosy, meaning that literate people had a lower risk, and illiterate people had a higher risk of TB and leprosy infection. The finding that literacy increases the risk of being notified positive for COVID-19 is possibly related to mobility (moving in and out of the neighbourhood) and being better informed about and having access to COVID-19 testing [18], whereas the finding that illiteracy increases the risk of TB and leprosy is likely related to poor knowledge of the disease, a lack of hygiene, lack of access to health care and poverty. Studies have also associated poverty-related factors such as illiteracy and poor water, sanitation and hygiene (WASH) conditions with TB and leprosy [19, 40, 41].

We also found that low monthly income (< minimum wage) and older age (≥ 65 years) had a negative effect on COVID-19 and a positive effect on TB. This implies that people with a higher income have an increased risk of COVID-19 and a decreased risk of TB; in other words, COVID-19 diagnosis is related to wealth, while TB is related to poverty. Especially at the beginning of the COVID-19 pandemic, COVID-19 cases were concentrated in neighbourhoods with the highest socioeconomic conditions (high income and literacy rate) [18]. The finding that people ≥ 65 years old have a lower risk of COVID-19 diagnosis may be because they were more socially isolated and protected by their family members after social distance guidelines were in place. In Brazil, older adults living with family members were associated with decreased COVID-19 testing. However, once they had symptoms, they were more likely to test for COVID-19 than those who live alone [42]. Older adults who were infected had a higher risk of developing severe COVID-19 and dying of COVID-19 due to the presence of comorbidities [43]. The higher risk of TB infection in the elderly is caused by the reactivation of lesions that have remained dormant and the senescence of the immune system [44].

The COVID-19 pandemic had a considerable impact on the health system in Fortaleza. Combined or integrated (preventive) interventions targeting the populations at high risk for COVID-19, TB and leprosy could reduce the burden on health workers in specific areas. A good example is the study of Banjara et al. (2015), which determined that a combined camp approach for vector control and active case detection of leishmaniasis, TB, leprosy and malaria was feasible, acceptable to health workers, and cost-effective [45]. For Fortaleza, neighbourhoods with high incidence rates of COVID-19 and TB (e.g., Praia de Iracema, Sabiaguaba and Manuel Dias Branco) could be targeted with vaccination campaigns (COVID-19 and Bacille Calmette-Guerin [BCG] vaccines) to strengthen the host’s immune system and prevent disease. However, the current BCG vaccine is only effective in children or if the first and/or second doses have not been given [46]. In neighbourhoods with low access to piped water and poor WASH conditions, large households, and low literacy (e.g., Cais do Porto, Genibau and Autran Nunes), education on good ventilation and good hygiene could prevent further spread of aerosols in these communities. In the more southern neighbourhoods, where we found high incidence rates of TB and leprosy, early case detection and administration of preventive treatment to contacts can be implemented to interrupt the transmission of *M. tuberculosis* and *M. leprae*. A combined community-wide active case finding approach consisting of screening for signs and symptoms of TB and leprosy, testing for TB infection and administering preventive treatment (i.e., 4R or 6 H and SDR- PEP) could be useful for the PHCs to allocate the available (human) resources more efficiently. Currently, a population-wide ‘screen and treat’ intervention, including screening, treatment and prevention for TB and leprosy, is being conducted in Kiribati to test the effect on TB and leprosy incidences and the feasibility of using combined preventive interventions [47]. Policymakers, however, should consider that COVID-19, TB and leprosy have different courses of disease and social perceptions. Either stigma related to leprosy and TB can be a barrier to the successful implementation of combined interventions, or combined interventions can reduce stigma as the diagnosis of TB or leprosy will not be disclosed [48].

In this study, all three models had a negative log-likelihood of more than 500, indicating a poor fit. This is likely caused by high multicollinearity and significant heteroskedasticity. We found strong correlations between the variables included in the models. Some of the variables reflect poverty and are, therefore, strongly correlated. This probably influenced the model, but not significantly. Moreover, a limitation of this study is the availability of recent census data. Due to the COVID-19 pandemic, the census survey planned for 2020 was cancelled, and only the data from 2010 was available. This makes interpreting our findings difficult as Fortaleza has expanded and developed in the past 12 years. For example, the eastern coast of Fortaleza is now a highly developed area with apartment buildings and restaurants. In our analysis, the area shows low income, low literacy and small population densities. The outdated data may have contributed to the poor fit of our models; as such, when more accurate data is available, we can improve the models.

## Conclusion

Despite the similar mode of transmission, COVID-19, TB and leprosy present a different distribution of cases in Fortaleza. In addition, associated risk factors are related to wealth in COVID-19 and to poverty in TB and leprosy. These findings can support policymakers in developing (combined) primary and secondary preventions with consideration of the efficient use of resources.

### Electronic supplementary material

Below is the link to the electronic supplementary material.

Supplementary Material 1: Table S1 Dataset; Table S2. Pearson’s correlation coefficients of the different variables.

## Data Availability

The dataset supporting the conclusions of this article is included within the article (S1 Table in Supporting Information file).

## Abbreviations

BCG: Bacille Calmette-Guerin
COVID-19: Coronavirus disease 2019
HDI: Human Development Index
LM: Lagrange Multiplier
OLS: Ordinary Least Squaresr
PHC: Primary Healthcare Center
PEP: Post-Exposure Prophylaxis
SEM: Spatial Error Model
SLM: Spatial Lag Model
TB: tuberculosis
WHO: World Health Organization
