# Supplementary material for: The geographical distribution and socioeconomic risk factors of COVID-19, tuberculosis and leprosy in Fortaleza, Brazil
Source: BMC Infect Dis. 2023 Oct 6;23:662. doi: 10.1186/s12879-023-08627-9 (PMC10585722; doi:10.1186/s12879-023-08627-9)
Supplement: Supplementary file 1 — Supplementary Material 1: Table S1 Dataset; Table S2. Pearson’s correlation coefficients of the different variables. [file 12879_2023_8627_MOESM1_ESM.docx]

Supporting information

S1 Table. Dataset

| **Name Neighbourhood** | **Population 2022** | **Area km2** | **Population density** | **Total COVID-19 cases** | **Rate COVID-19/100000** | **Total TB 2015-21** | **Rate TB/100000** | **Total Leprosy 2014-20** | **Rate Leprosy/100000** | **CENSUS 2010 Population** | **Mean HH size** | **Prop HH>4** | **Prop HH1-2** | **Prop HH3-5** | **Prop HH6** | **Mean monthly income** | **Prop <1wage** | **Prop >1wage** | **Prop own bath** | **Prop female** | **Prop age<15** | **Prop age15-64** | **Prop age>65** | **Prop literate** |
| --- | --- | --- | --- | --- | --- | --- | --- | --- | --- | --- | --- | --- | --- | --- | --- | --- | --- | --- | --- | --- | --- | --- | --- | --- |
| Aerolândia | 12523 | 1.114 | 10197 | 1637 | 13072 | 86 | 687 | 10 | 80 | 11360 | 3.52 | 0.45 | 0.30 | 0.59 | 0.11 | 486.27 | 0.74 | 0.26 | 1.00 | 0.53 | 0.21 | 0.71 | 0.08 | 0.87 |
| Aeroporto (Base Aérea) | 9501 | 5.564 | 1549 | 381 | 4010 | 9 | 95 | 7 | 74 | 8618 | 3.61 | 0.50 | 0.27 | 0.61 | 0.12 | 452.81 | 0.77 | 0.23 | 0.99 | 0.52 | 0.21 | 0.72 | 0.08 | 0.88 |
| Alagadiço | 15990 | 1.475 | 9834 | 1716 | 10732 | 10 | 63 | 10 | 63 | 14505 | 3.30 | 0.41 | 0.33 | 0.58 | 0.08 | 1351.90 | 0.28 | 0.71 | 0.99 | 0.56 | 0.25 | 0.70 | 0.06 | 0.84 |
| Aldeota | 46701 | 3.876 | 10930 | 10306 | 22068 | 129 | 276 | 13 | 28 | 42361 | 3.08 | 0.36 | 0.39 | 0.55 | 0.06 | 2915.77 | 0.15 | 0.85 | 1.00 | 0.58 | 0.26 | 0.70 | 0.04 | 0.83 |
| Alto da Balança | 14127 | 0.925 | 13853 | 904 | 6399 | 42 | 297 | 26 | 184 | 12814 | 3.41 | 0.42 | 0.33 | 0.57 | 0.10 | 498.67 | 0.72 | 0.28 | 0.99 | 0.54 | 0.27 | 0.68 | 0.04 | 0.78 |
| Álvaro Weyne | 26117 | 1.418 | 16707 | 2575 | 9859 | 130 | 498 | 14 | 54 | 23690 | 3.51 | 0.44 | 0.29 | 0.60 | 0.10 | 597.95 | 0.64 | 0.36 | 0.99 | 0.54 | 0.23 | 0.71 | 0.07 | 0.86 |
| Amadeo Furtado | 12901 | 0.928 | 12611 | 839 | 6503 | 9 | 70 | 8 | 62 | 11703 | 3.51 | 0.45 | 0.31 | 0.56 | 0.12 | 945.49 | 0.50 | 0.50 | 0.99 | 0.55 | 0.21 | 0.71 | 0.07 | 0.86 |
| Ancuri | 7418 | 4.113 | 4880 | 1321 | 17808 | 43 | 580 | 58 | 782 | 20070 | 3.34 | 0.42 | 0.31 | 0.59 | 0.09 | 465.76 | 0.82 | 0.18 | 0.98 | 0.53 | 0.23 | 0.71 | 0.07 | 0.86 |
| Antônio Bezerra | 28493 | 2.194 | 11780.31 | 4017 | 14098 | 131 | 460 | 25 | 88 | 25846 | 3.45 | 0.44 | 0.30 | 0.59 | 0.10 | 558.06 | 0.67 | 0.32 | 0.99 | 0.54 | 0.21 | 0.71 | 0.08 | 0.87 |
| Arraial Moura Brasil | 4150 | 0.472 | 7977 | 261 | 6289 | 31 | 747 | 2 | 48 | 3765 | 3.56 | 0.45 | 0.33 | 0.54 | 0.12 | 447.64 | 0.75 | 0.24 | 0.98 | 0.53 | 0.16 | 0.74 | 0.10 | 0.93 |
| Autran Nunes | 23380 | 0.988 | 21465 | 1322 | 5654 | 97 | 415 | 29 | 124 | 21208 | 3.78 | 0.51 | 0.24 | 0.61 | 0.14 | 355.73 | 0.87 | 0.13 | 1.00 | 0.52 | 0.19 | 0.72 | 0.09 | 0.89 |
| Barra do Ceará | 79842 | 4.292 | 16874 | 5901 | 7391 | 341 | 427 | 72 | 91 | 72423 | 3.57 | 0.46 | 0.29 | 0.59 | 0.12 | 395.10 | 0.82 | 0.17 | 0.99 | 0.52 | 0.25 | 0.67 | 0.08 | 0.80 |
| Barroso | 32905 | 3.49 | 8552 | 2599 | 7898 | 129 | 392 | 52 | 158 | 29847 | 3.60 | 0.47 | 0.27 | 0.61 | 0.12 | 399.92 | 0.83 | 0.17 | 0.99 | 0.52 | 0.20 | 0.70 | 0.10 | 0.88 |
| Bela Vista | 18470 | 0.973 | 17219 | 1918 | 10384 | 88 | 476 | 13 | 70 | 16754 | 3.39 | 0.42 | 0.34 | 0.57 | 0.09 | 660.12 | 0.63 | 0.37 | 1.00 | 0.54 | 0.22 | 0.71 | 0.07 | 0.84 |
| Benfica | 9867 | 0.958 | 9363 | 2277 | 23077 | 37 | 375 | 4 | 41 | 8970 | 3.01 | 0.32 | 0.42 | 0.49 | 0.06 | 1044.04 | 0.46 | 0.54 | 0.97 | 0.57 | 0.26 | 0.69 | 0.06 | 0.82 |
| Bom Futuro | 7060 | 0.384 | 16679 | 664 | 9405 | 7 | 99 | 3 | 42 | 6405 | 3.24 | 0.38 | 0.36 | 0.56 | 0.08 | 778.94 | 0.52 | 0.48 | 1.00 | 0.57 | 0.25 | 0.70 | 0.05 | 0.82 |
| Bom Jardim | 41626 | 2.406 | 15693 | 4502 | 10815 | 406 | 975 | 113 | 271 | 37758 | 3.60 | 0.47 | 0.28 | 0.60 | 0.12 | 350.23 | 0.86 | 0.14 | 0.99 | 0.52 | 0.24 | 0.69 | 0.07 | 0.82 |
| Bonsucesso | 45418 | 2.515 | 16381 | 3460 | 7618 | 179 | 394 | 91 | 200 | 41198 | 3.51 | 0.45 | 0.29 | 0.60 | 0.11 | 438.15 | 0.78 | 0.22 | 1.00 | 0.53 | 0.23 | 0.70 | 0.07 | 0.86 |
| Cais do Porto | 24674 | 3.095 | 7232 | 1169 | 4738 | 107 | 434 | 11 | 45 | 22382 | 3.56 | 0.45 | 0.29 | 0.59 | 0.12 | 400.57 | 0.84 | 0.16 | 0.99 | 0.51 | 0.18 | 0.72 | 0.10 | 0.89 |
| Cajazeiras | 15961 | 3.439 | 4210 | 1415 | 8865 | 23 | 144 | 15 | 94 | 14478 | 3.24 | 0.39 | 0.34 | 0.59 | 0.07 | 810.28 | 0.53 | 0.47 | 0.99 | 0.53 | 0.17 | 0.73 | 0.10 | 0.90 |
| Cambeba | 8405 | 2.753 | 2769 | 1837 | 21856 | 15 | 178 | 1 | 12 | 7625 | 3.51 | 0.50 | 0.25 | 0.67 | 0.07 | 1613.25 | 0.39 | 0.61 | 1.00 | 0.53 | 0.19 | 0.73 | 0.08 | 0.89 |
| Canindezinho | 45422 | 3.781 | 10897 | 2712 | 5971 | 146 | 321 | 102 | 225 | 41202 | 3.59 | 0.47 | 0.27 | 0.61 | 0.12 | 320.02 | 0.88 | 0.12 | 0.99 | 0.51 | 0.21 | 0.71 | 0.09 | 0.88 |
| Carlito Pamplona | 32055 | 1.352 | 21506 | 1753 | 5469 | 95 | 296 | 25 | 78 | 29076 | 3.48 | 0.44 | 0.31 | 0.58 | 0.11 | 515.64 | 0.71 | 0.29 | 1.00 | 0.53 | 0.13 | 0.74 | 0.12 | 0.95 |
| Castelão | 6571 | 1.949 | 3065 | 1958 | 29798 | 67 | 1020 | 11 | 167 | 5974 | 3.64 | 0.46 | 0.26 | 0.57 | 0.13 | 487.38 | 0.74 | 0.21 | 0.95 | 0.52 | 0.15 | 0.73 | 0.12 | 0.92 |
| Centro | 31463 | 4.89 | 5836 | 6575 | 20898 | 185 | 588 | 27 | 86 | 28538 | 2.87 | 0.31 | 0.47 | 0.44 | 0.06 | 1084.42 | 0.38 | 0.60 | 0.97 | 0.54 | 0.13 | 0.76 | 0.11 | 0.95 |
| Cidade 2000 | 9120 | 0.495 | 1671 | 1995 | 21875 | 46 | 504 | 5 | 55 | 8272 | 3.16 | 0.36 | 0.38 | 0.56 | 0.07 | 1027.54 | 0.36 | 0.64 | 1.00 | 0.57 | 0.13 | 0.73 | 0.14 | 0.94 |
| Cidade dos Funcionários | 20127 | 2.787 | 655 | 2326 | 11557 | 46 | 229 | 8 | 40 | 18256 | 3.39 | 0.44 | 0.31 | 0.59 | 0.09 | 1493.08 | 0.33 | 0.66 | 0.99 | 0.55 | 0.17 | 0.74 | 0.10 | 0.92 |
| Coaçu | 7924 | 1.707 | 4211 | 895 | 11295 | 10 | 126 | 4 | 50 | 7188 | 3.53 | 0.48 | 0.27 | 0.63 | 0.09 | 595.62 | 0.70 | 0.30 | 1.00 | 0.52 | 0.14 | 0.76 | 0.11 | 0.93 |
| Coco | 22590 | 3.247 | 6311 | 4147 | 18358 | 23 | 102 | 6 | 27 | 20492 | 3.24 | 0.41 | 0.35 | 0.59 | 0.06 | 3243.41 | 0.13 | 0.87 | 1.00 | 0.55 | 0.18 | 0.71 | 0.10 | 0.90 |
| Conjunto Ceará I | 21190 | 1.559 | 12329 | 5807 | 27404 | 81 | 382 | 14 | 66 | 19221 | 3.51 | 0.45 | 0.28 | 0.61 | 0.10 | 595.87 | 0.61 | 0.39 | 1.00 | 0.54 | 0.17 | 0.73 | 0.10 | 0.89 |
| Conjunto Ceará II | 26099 | 1.827 | 12957 | 858 | 3287 | 4 | 15 | 20 | 77 | 23673 | 3.51 | 0.45 | 0.28 | 0.62 | 0.10 | 581.45 | 0.62 | 0.38 | 1.00 | 0.54 | 0.15 | 0.74 | 0.11 | 0.93 |
| Conjunto Esperança | 18085 | 1.132 | 14492 | 1581 | 8742 | 35 | 194 | 40 | 221 | 16405 | 3.48 | 0.45 | 0.28 | 0.62 | 0.09 | 521.23 | 0.69 | 0.31 | 1.00 | 0.53 | 0.17 | 0.72 | 0.11 | 0.90 |
| Conjunto Palmeiras | 40347 | 7.639 | 4791 | 1989 | 4930 | 142 | 352 | 78 | 193 | 36599 | 3.97 | 0.51 | 0.26 | 0.57 | 0.18 | 262.83 | 0.93 | 0.07 | 0.99 | 0.51 | 0.14 | 0.73 | 0.13 | 0.95 |
| Couto Fernades | 5799 | 0.33 | 1593 | 392 | 6760 | 24 | 414 | 7 | 121 | 5260 | 3.35 | 0.42 | 0.34 | 0.57 | 0.09 | 672.63 | 0.65 | 0.35 | 1.00 | 0.53 | 0.10 | 0.80 | 0.10 | 0.95 |
| Cristo Redentor | 29454 | 1.168 | 2287 | 1984 | 6736 | 212 | 720 | 10 | 34 | 26717 | 3.71 | 0.49 | 0.27 | 0.59 | 0.14 | 380.57 | 0.83 | 0.17 | 0.99 | 0.53 | 0.14 | 0.74 | 0.12 | 0.93 |
| Curió | 8419 | 0.646 | 11820 | 693 | 8231 | 48 | 570 | 6 | 71 | 7636 | 3.58 | 0.50 | 0.26 | 0.64 | 0.09 | 520.72 | 0.77 | 0.23 | 1.00 | 0.51 | 0.17 | 0.71 | 0.12 | 0.91 |
| Damas | 11817 | 0.911 | 11766 | 1922 | 16265 | 27 | 228 | 4 | 34 | 10719 | 3.14 | 0.37 | 0.38 | 0.55 | 0.07 | 921.87 | 0.45 | 0.55 | 1.00 | 0.56 | 0.18 | 0.72 | 0.10 | 0.92 |
| De Lourdes | 3716 | 1.968 | 1712 | 474 | 12756 | 0 | 0 | 0 | 0 | 3370 | 3.46 | 0.44 | 0.32 | 0.57 | 0.11 | 2984.17 | 0.24 | 0.76 | 0.98 | 0.54 | 0.11 | 0.76 | 0.13 | 0.95 |
| Demócrito Rocha | 12119 | 0.803 | 13691 | 2103 | 17353 | 59 | 487 | 14 | 116 | 10994 | 3.38 | 0.43 | 0.34 | 0.57 | 0.09 | 549.67 | 0.71 | 0.29 | 1.00 | 0.54 | 0.25 | 0.68 | 0.07 | 0.80 |
| Dendê | 6215 | 1.941 | 2904 | 514 | 8270 | 12 | 193 | 15 | 241 | 5637 | 3.27 | 0.33 | 0.27 | 0.50 | 0.06 | 714.35 | 0.51 | 0.36 | 0.83 | 0.47 | 0.14 | 0.72 | 0.14 | 0.92 |
| Dias Macedo | 13353 | 1.833 | 6607 | 1276 | 9556 | 41 | 307 | 17 | 127 | 12111 | 3.49 | 0.45 | 0.29 | 0.60 | 0.10 | 418.46 | 0.79 | 0.21 | 0.99 | 0.53 | 0.16 | 0.76 | 0.08 | 0.92 |
| Dom Lustosa | 14495 | 1.204 | 10919 | 758 | 5229 | 25 | 172 | 27 | 186 | 13147 | 3.44 | 0.43 | 0.30 | 0.59 | 0.10 | 566.16 | 0.68 | 0.32 | 1.00 | 0.54 | 0.22 | 0.72 | 0.06 | 0.87 |
| Edson Queiroz | 24485 | 14.06 | 1579 | 3072 | 12546 | 111 | 453 | 8 | 33 | 22210 | 3.78 | 0.52 | 0.26 | 0.58 | 0.16 | 863.15 | 0.64 | 0.36 | 1.00 | 0.52 | 0.21 | 0.72 | 0.07 | 0.88 |
| Engenheiro Luciano Cavalcante | 17134 | 3.873 | 4013 | 3529 | 20596 | 69 | 403 | 16 | 93 | 15543 | 3.50 | 0.44 | 0.31 | 0.58 | 0.11 | 1407.81 | 0.48 | 0.52 | 0.99 | 0.54 | 0.23 | 0.73 | 0.04 | 0.86 |
| Estância (Dionísio Torres) | 17235 | 1.747 | 8949 | 3030 | 17581 | 17 | 99 | 5 | 29 | 15634 | 3.28 | 0.42 | 0.33 | 0.60 | 0.07 | 2511.63 | 0.16 | 0.83 | 1.00 | 0.57 | 0.25 | 0.70 | 0.05 | 0.84 |
| Farias Brito | 13299 | 0.941 | 12819 | 1301 | 9783 | 57 | 429 | 6 | 45 | 12063 | 3.38 | 0.41 | 0.34 | 0.55 | 0.10 | 855.92 | 0.49 | 0.50 | 0.99 | 0.55 | 0.25 | 0.70 | 0.06 | 0.81 |
| Fátima | 25697 | 2.869 | 8124 | 4679 | 18208 | 52 | 202 | 19 | 74 | 23309 | 3.21 | 0.39 | 0.35 | 0.56 | 0.07 | 1802.58 | 0.21 | 0.78 | 0.98 | 0.57 | 0.24 | 0.72 | 0.03 | 0.84 |
| Floresta | 31855 | 1.698 | 17017 | 883 | 2772 | 68 | 213 | 31 | 97 | 28896 | 3.51 | 0.45 | 0.30 | 0.59 | 0.11 | 380.91 | 0.83 | 0.17 | 1.00 | 0.53 | 0.17 | 0.75 | 0.08 | 0.92 |
| Genibau | 44466 | 2.148 | 18778 | 2310 | 5195 | 143 | 322 | 117 | 263 | 40336 | 3.55 | 0.46 | 0.28 | 0.61 | 0.11 | 333.54 | 0.87 | 0.13 | 0.99 | 0.52 | 0.18 | 0.74 | 0.08 | 0.91 |
| Gentilândia | 4382 | 0.484 | 8231 | 0 | 15952 | 0 | 0 | 3 | 68 | 3984 | 3.13 | 0.36 | 0.39 | 0.51 | 0.07 | 1339.99 | 0.29 | 0.70 | 0.98 | 0.57 | 0.19 | 0.74 | 0.06 | 0.88 |
| Granja Lisboa | 57373 | 5.453 | 9543 | 2898 | 5051 | 138 | 241 | 164 | 286 | 52042 | 3.62 | 0.48 | 0.28 | 0.60 | 0.12 | 339.21 | 0.87 | 0.13 | 0.99 | 0.51 | 0.21 | 0.74 | 0.05 | 0.89 |
| Granja Portugal | 43714 | 2.528 | 15684 | 3331 | 7620 | 298 | 682 | 161 | 368 | 39651 | 3.68 | 0.49 | 0.26 | 0.61 | 0.13 | 329.20 | 0.87 | 0.13 | 0.98 | 0.52 | 0.24 | 0.69 | 0.08 | 0.84 |
| Guajeru | 7350 | 1.076 | 6197 | 528 | 7184 | 21 | 286 | 5 | 68 | 6668 | 3.62 | 0.48 | 0.25 | 0.64 | 0.11 | 565.19 | 0.70 | 0.30 | 1.00 | 0.54 | 0.26 | 0.69 | 0.05 | 0.81 |
| Guarapes | 5805 | 1.343 | 3921 | 1556 | 26804 | 7 | 121 | 5 | 86 | 5266 | 3.38 | 0.47 | 0.31 | 0.61 | 0.08 | 3308.53 | 0.14 | 0.86 | 1.00 | 0.55 | 0.22 | 0.73 | 0.06 | 0.86 |
| Henrique Jorge | 29761 | 1.944 | 13886 | 3559 | 11959 | 104 | 349 | 42 | 141 | 26994 | 3.45 | 0.44 | 0.30 | 0.60 | 0.10 | 556.71 | 0.69 | 0.31 | 0.99 | 0.53 | 0.24 | 0.70 | 0.06 | 0.84 |
| Itaóca | 13754 | 0.736 | 16952 | 1430 | 10397 | 65 | 473 | 14 | 102 | 12477 | 3.34 | 0.41 | 0.33 | 0.58 | 0.09 | 608.33 | 0.63 | 0.37 | 1.00 | 0.55 | 0.28 | 0.68 | 0.04 | 0.80 |
| Itaperi | 24874 | 2.531 | 8915 | 3431 | 13794 | 83 | 334 | 22 | 88 | 22563 | 3.16 | 0.36 | 0.37 | 0.56 | 0.07 | 807.46 | 0.52 | 0.48 | 1.00 | 0.54 | 0.19 | 0.74 | 0.08 | 0.90 |
| Jacarecanga | 15658 | 1.304 | 10893 | 2596 | 16579 | 178 | 1137 | 6 | 38 | 14204 | 3.36 | 0.40 | 0.31 | 0.55 | 0.09 | 759.09 | 0.55 | 0.41 | 0.96 | 0.55 | 0.28 | 0.66 | 0.05 | 0.79 |
| Jangurussu | 55652 | 6.244 | 8084 | 7389 | 13277 | 243 | 437 | 72 | 129 | 50479 | 3.56 | 0.46 | 0.29 | 0.59 | 0.11 | 428.35 | 0.77 | 0.23 | 0.99 | 0.52 | 0.29 | 0.67 | 0.04 | 0.78 |
| Jardim América | 13520 | 0.769 | 15948 | 1540 | 11391 | 64 | 473 | 8 | 59 | 12264 | 3.38 | 0.43 | 0.32 | 0.58 | 0.09 | 710.68 | 0.57 | 0.43 | 1.00 | 0.56 | 0.29 | 0.68 | 0.03 | 0.80 |
| Jardim Cearense | 11138 | 0.871 | 11599 | 988 | 8871 | 11 | 99 | 11 | 99 | 10103 | 3.47 | 0.46 | 0.29 | 0.61 | 0.09 | 701.10 | 0.59 | 0.41 | 0.99 | 0.53 | 0.19 | 0.74 | 0.07 | 0.90 |
| Jardim das Oliveiras | 32599 | 2.447 | 12085 | 2709 | 8310 | 130 | 399 | 28 | 86 | 29571 | 3.55 | 0.46 | 0.29 | 0.59 | 0.12 | 457.44 | 0.79 | 0.21 | 1.00 | 0.53 | 0.28 | 0.69 | 0.03 | 0.78 |
| Jardim Guanabara | 16447 | 0.734 | 20325 | 1690 | 10275 | 56 | 340 | 3 | 18 | 14919 | 3.48 | 0.43 | 0.30 | 0.60 | 0.10 | 515.87 | 0.71 | 0.29 | 1.00 | 0.53 | 0.20 | 0.74 | 0.06 | 0.89 |
| Jardim Iracema | 25559 | 1.099 | 21095 | 2257 | 8831 | 89 | 348 | 22 | 86 | 23184 | 3.55 | 0.45 | 0.30 | 0.59 | 0.11 | 441.72 | 0.77 | 0.23 | 0.99 | 0.53 | 0.25 | 0.71 | 0.05 | 0.83 |
| João XXIII | 20283 | 1.166 | 15779 | 2300 | 11340 | 58 | 286 | 16 | 79 | 18398 | 3.52 | 0.45 | 0.29 | 0.60 | 0.11 | 457.01 | 0.75 | 0.25 | 0.99 | 0.53 | 0.27 | 0.70 | 0.04 | 0.81 |
| Joaquim Távora | 25854 | 1.976 | 1186 | 4462 | 17258 | 52 | 201 | 8 | 31 | 23450 | 3.18 | 0.38 | 0.37 | 0.55 | 0.07 | 1482.32 | 0.32 | 0.67 | 0.99 | 0.57 | 0.25 | 0.68 | 0.07 | 0.80 |
| Jóquei Club (São Cristóvão) | 21310 | 1.706 | 11331 | 2665 | 12506 | 59 | 277 | 19 | 89 | 19331 | 3.42 | 0.42 | 0.33 | 0.57 | 0.10 | 723.60 | 0.57 | 0.42 | 1.00 | 0.54 | 0.26 | 0.69 | 0.05 | 0.81 |
| José Bonifácio | 9754 | 0.888 | 9964 | 1380 | 14148 | 25 | 256 | 3 | 31 | 8848 | 3.14 | 0.35 | 0.39 | 0.51 | 0.08 | 1121.81 | 0.33 | 0.66 | 0.99 | 0.57 | 0.23 | 0.73 | 0.04 | 0.85 |
| José de Alencar | 17643 | 3.118 | 5132 | 1638 | 9284 | 26 | 147 | 10 | 57 | 16003 | 3.51 | 0.47 | 0.28 | 0.63 | 0.09 | 1248.74 | 0.54 | 0.46 | 1.00 | 0.52 | 0.24 | 0.72 | 0.04 | 0.79 |
| Lagoa Redonda | 30811 | 11.601 | 2409 | 2879 | 9344 | 78 | 253 | 46 | 149 | 27949 | 3.56 | 0.46 | 0.28 | 0.61 | 0.11 | 512.71 | 0.76 | 0.24 | 0.99 | 0.52 | 0.28 | 0.68 | 0.04 | 0.80 |
| Lagoa Sapiranga (Coité) | 35452 | 4.742 | 6781 | 3263 | 9204 | 96 | 271 | 18 | 51 | 32158 | 3.71 | 0.51 | 0.25 | 0.63 | 0.13 | 885.97 | 0.68 | 0.32 | 0.99 | 0.53 | 0.20 | 0.72 | 0.08 | 0.88 |
| Manoel Sátiro | 41841 | 3.04 | 12484 | 1915 | 4577 | 43 | 103 | 66 | 158 | 37952 | 3.48 | 0.43 | 0.30 | 0.61 | 0.09 | 540.91 | 0.69 | 0.29 | 1.00 | 0.54 | 0.26 | 0.69 | 0.04 | 0.83 |
| Manuel Dias Branco | 1593 | 4.294 | 337 | 702 | 44068 | 15 | 942 | 0 | 0 | 1447 | 3.36 | 0.45 | 0.32 | 0.58 | 0.10 | 1064.65 | 0.67 | 0.33 | 0.97 | 0.53 | 0.26 | 0.70 | 0.03 | 0.84 |
| Maraponga | 11197 | 1.62 | 6268 | 3426 | 30597 | 35 | 313 | 11 | 98 | 10155 | 3.32 | 0.41 | 0.32 | 0.59 | 0.08 | 850.91 | 0.51 | 0.49 | 1.00 | 0.53 | 0.24 | 0.72 | 0.04 | 0.84 |
| Mata Galinha | 6900 | 1.065 | 5890 | 0 | 14188 | 0 | 0 | 7 | 101 | 6273 | 3.27 | 0.39 | 0.34 | 0.57 | 0.08 | 700.25 | 0.59 | 0.40 | 0.98 | 0.52 | 0.27 | 0.69 | 0.04 | 0.82 |
| Meireles | 40770 | 2.726 | 13566 | 10463 | 25663 | 67 | 164 | 9 | 22 | 36982 | 2.87 | 0.31 | 0.47 | 0.47 | 0.05 | 3665.77 | 0.13 | 0.87 | 1.00 | 0.56 | 0.23 | 0.72 | 0.05 | 0.86 |
| Messejana (sede) | 45960 | 6.053 | 688 | 7795 | 16960 | 167 | 363 | 54 | 117 | 41689 | 3.42 | 0.43 | 0.31 | 0.59 | 0.10 | 641.00 | 0.62 | 0.37 | 0.99 | 0.54 | 0.15 | 0.73 | 0.12 | 0.93 |
| Mondubim (Sede) | 83832 | 9.318 | 8161 | 7393 | 8819 | 170 | 203 | 81 | 98 | 76044 | 3.42 | 0.44 | 0.30 | 0.62 | 0.09 | 519.91 | 0.72 | 0.28 | 1.00 | 0.52 | 0.13 | 0.75 | 0.13 | 0.94 |
| Monte Castelo | 14569 | 0.784 | 16856 | 2435 | 16714 | 65 | 446 | 15 | 103 | 13215 | 3.45 | 0.45 | 0.32 | 0.58 | 0.10 | 699.39 | 0.57 | 0.43 | 1.00 | 0.54 | 0.19 | 0.73 | 0.08 | 0.87 |
| Montese | 28630 | 2.132 | 12181 | 4715 | 16469 | 115 | 402 | 30 | 105 | 25970 | 3.26 | 0.40 | 0.36 | 0.56 | 0.08 | 833.88 | 0.50 | 0.50 | 1.00 | 0.56 | 0.26 | 0.69 | 0.05 | 0.79 |
| Mucuripe | 15155 | 0.875 | 15711 | 2144 | 14147 | 51 | 337 | 3 | 20 | 13747 | 3.03 | 0.35 | 0.43 | 0.51 | 0.06 | 3101.16 | 0.28 | 0.72 | 0.99 | 0.54 | 0.26 | 0.70 | 0.04 | 0.82 |
| Padre Andrade (Cachoeirinha) | 14263 | 1.255 | 10307 | 1050 | 7362 | 50 | 351 | 7 | 49 | 12936 | 3.43 | 0.43 | 0.30 | 0.58 | 0.10 | 613.22 | 0.64 | 0.34 | 0.98 | 0.54 | 0.19 | 0.75 | 0.06 | 0.89 |
| Pan-Americano | 9719 | 0.552 | 15969 | 1089 | 11205 | 50 | 514 | 14 | 144 | 8815 | 3.41 | 0.42 | 0.31 | 0.59 | 0.10 | 587.75 | 0.63 | 0.37 | 0.99 | 0.54 | 0.23 | 0.71 | 0.06 | 0.83 |
| Papicu | 20254 | 1.517 | 12109 | 4331 | 21383 | 172 | 849 | 9 | 44 | 18370 | 3.29 | 0.40 | 0.36 | 0.55 | 0.08 | 1579.73 | 0.43 | 0.57 | 0.99 | 0.54 | 0.29 | 0.68 | 0.03 | 0.78 |
| Parangaba | 34118 | 4.121 | 7509 | 4456 | 13061 | 111 | 325 | 34 | 100 | 30947 | 3.37 | 0.40 | 0.31 | 0.57 | 0.09 | 759.16 | 0.52 | 0.45 | 0.97 | 0.54 | 0.12 | 0.75 | 0.12 | 0.94 |
| Parque Araxá | 7403 | 0.462 | 14535 | 981 | 13251 | 22 | 297 | 6 | 81 | 6715 | 3.30 | 0.39 | 0.36 | 0.54 | 0.09 | 970.17 | 0.41 | 0.58 | 0.99 | 0.57 | 0.27 | 0.69 | 0.05 | 0.78 |
| Parque Dois Irmãos | 30025 | 4.452 | 6118 | 2593 | 8636 | 95 | 316 | 31 | 103 | 27236 | 3.69 | 0.50 | 0.25 | 0.64 | 0.11 | 497.86 | 0.73 | 0.27 | 0.99 | 0.52 | 0.15 | 0.74 | 0.10 | 0.91 |
| Parque Iracema | 9271 | 1.566 | 5369 | 1274 | 13742 | 12 | 129 | 8 | 86 | 8409 | 3.17 | 0.37 | 0.38 | 0.55 | 0.07 | 1530.83 | 0.34 | 0.66 | 1.00 | 0.54 | 0.17 | 0.75 | 0.08 | 0.93 |
| Parque Manibura | 8300 | 1.262 | 5966 | 869 | 10470 | 12 | 145 | 5 | 60 | 7529 | 3.73 | 0.53 | 0.23 | 0.65 | 0.12 | 1589.42 | 0.33 | 0.67 | 1.00 | 0.54 | 0.14 | 0.76 | 0.10 | 0.93 |
| Parque Presidente Vargas | 7929 | 1.553 | 4631 | 737 | 9295 | 31 | 391 | 30 | 378 | 7192 | 3.69 | 0.50 | 0.26 | 0.61 | 0.13 | 286.57 | 0.89 | 0.11 | 0.97 | 0.51 | 0.25 | 0.70 | 0.05 | 0.83 |
| Parque Santa Rosa (Apolo XI) | 14101 | 1.004 | 12739 | 1020 | 7234 | 26 | 184 | 26 | 184 | 12790 | 3.42 | 0.44 | 0.30 | 0.61 | 0.09 | 429.40 | 0.80 | 0.20 | 0.99 | 0.52 | 0.19 | 0.73 | 0.08 | 0.89 |
| Parque São José | 11561 | 0.602 | 17418 | 998 | 8632 | 51 | 441 | 31 | 268 | 10486 | 3.47 | 0.44 | 0.30 | 0.59 | 0.11 | 422.42 | 0.78 | 0.22 | 1.00 | 0.52 | 0.22 | 0.72 | 0.07 | 0.84 |
| Parquelândia | 15913 | 1.246 | 11582 | 3049 | 19160 | 55 | 346 | 5 | 31 | 14432 | 3.23 | 0.38 | 0.37 | 0.51 | 0.09 | 1168.36 | 0.36 | 0.62 | 0.97 | 0.56 | 0.20 | 0.71 | 0.09 | 0.88 |
| Parreão | 12207 | 1.138 | 9729 | 793 | 6496 | 11 | 90 | 5 | 41 | 11072 | 3.43 | 0.45 | 0.30 | 0.59 | 0.09 | 1227.80 | 0.33 | 0.65 | 0.98 | 0.55 | 0.21 | 0.72 | 0.08 | 0.87 |
| Passaré | 56158 | 7.214 | 7061 | 6528 | 11624 | 187 | 333 | 54 | 96 | 50940 | 3.41 | 0.42 | 0.31 | 0.59 | 0.09 | 626.12 | 0.68 | 0.32 | 0.99 | 0.52 | 0.20 | 0.74 | 0.06 | 0.88 |
| Paupina | 16166 | 5.052 | 2903 | 1823 | 11277 | 86 | 532 | 14 | 87 | 14665 | 3.50 | 0.45 | 0.27 | 0.63 | 0.09 | 474.79 | 0.78 | 0.22 | 0.99 | 0.52 | 0.23 | 0.71 | 0.06 | 0.85 |
| Pedras | 1479 | 7.117 | 188 | 867 | 58621 | 35 | 2366 | 4 | 270 | 1342 | 3.59 | 0.47 | 0.24 | 0.67 | 0.10 | 427.85 | 0.80 | 0.20 | 0.98 | 0.50 | 0.20 | 0.72 | 0.08 | 0.88 |
| Pici (Parque Universitário) | 46846 | 3.764 | 11289 | 2572 | 5490 | 156 | 333 | 28 | 60 | 42494 | 3.59 | 0.47 | 0.27 | 0.61 | 0.12 | 419.86 | 0.83 | 0.17 | 1.00 | 0.52 | 0.19 | 0.73 | 0.08 | 0.89 |
| Pirambú | 19596 | 0.567 | 31349 | 759 | 3873 | 196 | 1000 | 7 | 36 | 17775 | 3.67 | 0.47 | 0.27 | 0.59 | 0.13 | 346.27 | 0.86 | 0.14 | 0.98 | 0.53 | 0.30 | 0.66 | 0.04 | 0.85 |
| Planalto Ayrton Senna | 43488 | 4.181 | 9434 | 2148 | 4939 | 87 | 200 | 54 | 124 | 39446 | 3.55 | 0.47 | 0.28 | 0.60 | 0.11 | 371.22 | 0.84 | 0.16 | 0.99 | 0.51 | 0.23 | 0.72 | 0.05 | 0.85 |
| Praia de Iracema | 3452 | 0.529 | 5917 | 981 | 28418 | 37 | 1072 | 2 | 58 | 3130 | 2.80 | 0.27 | 0.53 | 0.41 | 0.06 | 2082.40 | 0.24 | 0.75 | 1.00 | 0.54 | 0.21 | 0.70 | 0.09 | 0.86 |
| Praia do Futuro I | 7310 | 2.137 | 3102 | 726 | 9932 | 70 | 958 | 2 | 27 | 6630 | 3.37 | 0.42 | 0.35 | 0.53 | 0.11 | 1024.92 | 0.63 | 0.37 | 0.97 | 0.52 | 0.23 | 0.71 | 0.06 | 0.84 |
| Praia do Futuro II | 13182 | 3.631 | 3293 | 1165 | 8838 | 1 | 8 | 9 | 68 | 11957 | 3.42 | 0.43 | 0.33 | 0.57 | 0.09 | 582.18 | 0.74 | 0.26 | 0.99 | 0.50 | 0.20 | 0.72 | 0.08 | 0.88 |
| Prefeito José Walter | 36853 | 11.232 | 2976 | 5413 | 14688 | 129 | 350 | 29 | 79 | 33427 | 3.41 | 0.43 | 0.32 | 0.58 | 0.09 | 632.79 | 0.61 | 0.39 | 0.99 | 0.54 | 0.18 | 0.72 | 0.10 | 0.89 |
| Presidente Kennedy | 25360 | 1.723 | 13351 | 2480 | 9779 | 77 | 304 | 13 | 51 | 23004 | 3.47 | 0.44 | 0.29 | 0.60 | 0.10 | 785.28 | 0.56 | 0.43 | 0.99 | 0.55 | 0.20 | 0.71 | 0.09 | 0.87 |
| Quintino Cunha | 52120 | 2.801 | 16878 | 2815 | 5401 | 207 | 397 | 60 | 115 | 47277 | 3.59 | 0.47 | 0.27 | 0.62 | 0.11 | 419.17 | 0.80 | 0.20 | 0.99 | 0.52 | 0.20 | 0.71 | 0.09 | 0.89 |
| Rodolfo Teófilo | 21071 | 1.696 | 11270 | 2668 | 12662 | 81 | 384 | 15 | 71 | 19114 | 3.37 | 0.42 | 0.33 | 0.56 | 0.10 | 816.27 | 0.52 | 0.48 | 0.99 | 0.55 | 0.26 | 0.70 | 0.04 | 0.81 |
| Sabiaguaba | 2334 | 10.192 | 208 | 585 | 25064 | 18 | 771 | 0 | 0 | 2117 | 3.56 | 0.48 | 0.27 | 0.61 | 0.11 | 539.71 | 0.78 | 0.21 | 0.99 | 0.50 | 0.21 | 0.72 | 0.08 | 0.87 |
| Salinas | 4737 | 2.475 | 1736 | 518 | 10935 | 7 | 148 | 1 | 21 | 4298 | 3.52 | 0.48 | 0.28 | 0.65 | 0.08 | 1887.82 | 0.37 | 0.63 | 0.99 | 0.53 | 0.22 | 0.73 | 0.05 | 0.88 |
| São Bento | 13189 | 2.699 | 4433 | 481 | 3647 | 14 | 106 | 8 | 61 | 11964 | 3.34 | 0.41 | 0.31 | 0.61 | 0.08 | 445.62 | 0.77 | 0.22 | 0.99 | 0.52 | 0.22 | 0.73 | 0.05 | 0.89 |
| São João do Tauapé | 30426 | 2.362 | 11684 | 3159 | 10383 | 140 | 460 | 37 | 122 | 27598 | 3.33 | 0.40 | 0.34 | 0.56 | 0.10 | 923.23 | 0.56 | 0.44 | 1.00 | 0.55 | 0.30 | 0.66 | 0.04 | 0.79 |
| Serrinha | 31715 | 3.182 | 9041 | 3522 | 11105 | 144 | 454 | 40 | 129 | 28770 | 3.47 | 0.44 | 0.30 | 0.60 | 0.10 | 537.07 | 0.72 | 0.28 | 0.99 | 0.52 | 0.27 | 0.70 | 0.03 | 0.83 |
| Siqueira | 37075 | 8.846 | 3801 | 3360 | 9063 | 117 | 316 | 131 | 353 | 33628 | 3.62 | 0.48 | 0.27 | 0.62 | 0.12 | 331.21 | 0.87 | 0.13 | 0.99 | 0.51 | 0.25 | 0.72 | 0.04 | 0.86 |
| Varjota | 9284 | 0.518 | 16257 | 1507 | 16232 | 37 | 399 | 4 | 43 | 8421 | 3.01 | 0.33 | 0.42 | 0.52 | 0.06 | 2170.00 | 0.24 | 0.76 | 1.00 | 0.56 | 0.28 | 0.68 | 0.04 | 0.80 |
| Vicente Pinzon | 50182 | 3.266 | 13937 | 4296 | 8561 | 243 | 484 | 20 | 40 | 45518 | 3.56 | 0.45 | 0.29 | 0.59 | 0.12 | 821.86 | 0.68 | 0.32 | 0.99 | 0.53 | 0.22 | 0.75 | 0.02 | 0.94 |
| Vila Ellery | 8668 | 0.467 | 16837 | 1270 | 14652 | 41 | 473 | 7 | 81 | 7863 | 3.44 | 0.44 | 0.30 | 0.61 | 0.09 | 682.24 | 0.58 | 0.42 | 1.00 | 0.55 | 0.20 | 0.71 | 0.09 | 0.90 |
| Vila Pery | 22760 | 1.502 | 13745 | 2396 | 10527 | 74 | 325 | 25 | 110 | 20645 | 3.39 | 0.42 | 0.32 | 0.59 | 0.09 | 521.95 | 0.70 | 0.30 | 1.00 | 0.54 | 0.26 | 0.70 | 0.04 | 0.85 |
| Vila União | 16953 | 1.41 | 10906 | 2397 | 14139 | 65 | 383 | 15 | 88 | 15378 | 3.42 | 0.42 | 0.30 | 0.59 | 0.09 | 913.76 | 0.52 | 0.46 | 0.98 | 0.54 | 0.21 | 0.73 | 0.05 | 0.87 |
| Vila Velha | 67930 | 7.221 | 8533 | 4020 | 5918 | 159 | 234 | 62 | 91 | 61617 | 3.55 | 0.47 | 0.28 | 0.61 | 0.11 | 489.82 | 0.74 | 0.26 | 1.00 | 0.53 | 0.24 | 0.70 | 0.05 | 0.84 |

**Table S2**. Pearson’s correlation coefficients of the different variables.

|  | Household size 1-2 person(s) | Household size 3-5 persons | Household size ≥ 6 persons | Monthly income ≥ minimum wage | Monthly income < minimum wage | Own bathroom & toilet | Female | Age < 15 years | Age 15 - 64 years | Age ≥ 65 years | Proportion literacy |
| --- | --- | --- | --- | --- | --- | --- | --- | --- | --- | --- | --- |
| Household size 1-2 person(s) |  | -1.077** | 1.596** | 0.163* | -0.161** | 0.146 | 1.676** | -0.765** | 1.155** | 1.165** | 0.638** |
| Household size 3-5 persons | -0.704** | - | 0.761** | -0.090** | 0.093** | -0.663** | 0.885** | -0.481** | -0.645** | -0.793** | -0.414** |
| Household size ≥ 6 persons | -0.305** | 0.223** | - | -0.078** | 0.078** | 0.133 | -0.614** | 0.314** | -0.616** | -0.370** | -0.310** |
| Monthly income ≥ minimum wage | 2.683** | -2.256** | -6.695** | - | -0.990** | 0.717 | 8.001** | -3.784** | 6.708** | 4.990** | 3.656** |
| Monthly income < minimum wage | -2.683** | 2.363** | 6.832** | -1.006** | - | 0.038 | -7.862** | 3.807** | -6.785** | -4.992** | -3.735** |
| Own bathroom & toilet | 0.018 | 0.123** | 0.084 | 0.005 | 0.000 | - | 0.307** | -0.035 | 0.004 | 0.090 | -0.021 |
| Female | 0.232** | -0.187** | -0.444** | 0.067** | -0.065** | 0.351** | - | -0.349** | 0.465** | 0.579** | 0.302** |
| Age < 15 years | -0.646** | 0..621** | 1.386** | -0.194** | 0.193** | -0.241 | -2.133** | - | -1.591** | -1.457** | -0.903** |
| Age 15 - 64 years | 0.280** | -0.239** | -0.780** | 0.009** | -0.098** | 0.008 | 0.814** | -0.457** | - | 0.456** | 0.442** |
| Age ≥ 65 years | 0.367** | -0.381** | -0.608** | 0.096** | -0.094** | 0.235 | 1.319** | -0.543** | 0.592** | - | 0.461** |
| Proportion literacy | 0.574** | -0.569** | -1.460** | 0.200** | -0.201** | -0.157 | 1.964** | -0.963** | 1.641** | 1.319** | - |

* p < 0.05, ** p < 0.01, *** p < 0.001
